# Nitrogen fertilizer rate but not form affects the severity of Fusarium wilt in banana

**DOI:** 10.3389/fpls.2022.907819

**Published:** 2022-07-22

**Authors:** Ryan Orr, Paul G. Dennis, Yide Wong, Daniel J. Browne, Martha Cooper, Henry W. G. Birt, Hazel R. Lapis-Gaza, Anthony B. Pattison, Paul N. Nelson

**Affiliations:** ^1^College of Science and Engineering, James Cook University, Cairns, QLD, Australia; ^2^School of Earth and Environmental Sciences, The University of Queensland, Brisbane, QLD, Australia; ^3^Australian Institute of Tropical Health and Medicine (AITHM), James Cook University, Cairns, QLD, Australia; ^4^Centre for Tropical Bioinformatics and Molecular Biology, James Cook University, Cairns, QLD, Australia; ^5^Centre for Molecular Therapeutics, James Cook University, Cairns, QLD, Australia; ^6^Department of Plant Science, The Pennsylvania State University, University Park, PA, United States; ^7^Huck Institutes of the Life Sciences, The Pennsylvania State University, University Park, PA, United States; ^8^Department of Agriculture and Fisheries, South Johnstone, QLD, Australia

**Keywords:** disease triangle, proteomics, ammonium, nitrate, qPCR, banana, nitrogen fertilisation, Fusarium wilt (causal agent *Fusarium oxysporum*)

## Abstract

Nitrogen (N) fertilizers are routinely applied to bananas (*Musa* spp.) to increase production but may exacerbate plant diseases like Fusarium wilt of banana (FWB), which is the most economically important disease. Here, we characterized the effects of N rate and form on banana plant growth, root proteome, bacterial and fungal diversity in the rhizosphere, the concentration of *Fusarium oxysporum* f.sp. *cubense* (Foc) in the soil, and the FWB severity. Banana plants (*Musa* subgroup ABB) were grown under greenhouse conditions in soil with ammonium or nitrate supplemented at five N rates, and with or without inoculation with Foc. The growth of non-inoculated plants was positively correlated with the N rate. In bananas inoculated with Foc, disease severity increased with the N rate, resulting in the Foc-inoculated plant growth being greatest at intermediate N rates. The abundance of Foc in the soil was weakly related to the treatment conditions and was a poor predictor of disease severity. Fungal diversity was consistently affected by Foc inoculation, while bacterial diversity was associated with changes in soil pH resulting from N addition, in particular ammonium. N rate altered the expression of host metabolic pathways associated with carbon fixation, energy usage, amino acid metabolism, and importantly stress response signaling, irrespective of inoculation or N form. Furthermore, in diseased plants, Pathogenesis-related protein 1, a key endpoint for biotic stress response and the salicylic acid defense response to biotrophic pathogens, was negatively correlated with the rate of ammonium fertilizer but not nitrate. As expected, inoculation with Foc altered the expression of a wide range of processes in the banana plant including those of defense and growth. In summary, our results indicate that the severity of FWB was negatively associated with host defenses, which was influenced by N application (particularly ammonium), and shifts in microbial communities associated with ammonium-induced acidification.

## Introduction

The application of synthetic nitrogen (N) fertilizer has enabled humans to dramatically increase agricultural productivity (Erisman et al., 2008). Worldwide use of synthetic N fertilizer is projected to increase to 112 million tonnes in 2022 (FAO, 2019). The use of N fertilizer has substantial benefits, but also some detrimental effects. Excessive N use has been found to increase the severity of diseases such as Fusarium wilt of banana (FWB) (Pittaway et al., 1999; Mur et al., 2017; Segura-Mena et al., 2021). Disease severity is governed by the three-way relationship between host, pathogen and the environment (Agrios, 2005), with N supply an especially important environmental aspect in the context of FWB.

Banana farmers apply N fertilizer to maximize productivity, potentially rendering their plants vulnerable to pathogens such as *F. oxysporum* f.sp. *cubense* (Foc), the soil-borne, hemibiotrophic, fungal pathogen responsible for FWB. The association between N fertilization and increased FWB severity remains unclear, as N fertilizers have multiple effects on soils, including changes to nutrient availability, porosity, and pH (Marinari et al., 2000; Neumann and Römheld, 2012). The main forms of N fertilizer used are ammonium, urea, and nitrate, with ammonium and urea being most common due to their lower price. Current evidence indicates that nitrate fertilizer reduces Fusarium wilt severity, whereas ammonium increases it (Woltz and Engelhard, 1973; Jones et al., 1975; Morgan and Timmer, 1984; Woltz et al., 1992; Wang et al., 2016; Zhou et al., 2017) though there are exceptions (Jarvis and Thorpe, 1980). This effect of N form on disease may be due to the acidifying effect of ammonium fertilizer and alkalizing effect of nitrate fertilizer, as soil acidification increases FWB severity (Orr and Nelson, 2018; Teixeira et al., 2021; Segura et al., 2022). Indeed, field trials have sown a greater influence of N fertilizer form on FWB severity at low pH than at high (Jones et al., 1975). Segura-Mena et al. (2021) found that the addition of ammonium nitrate fertilizer increased FWB severity, but less so at pH 5.1 than at 6.1. Only one soil-based study, performed on tomatoes, maintained similar pH between different N fertilizer forms and found nitrate to be significantly more suppressive to disease than ammonium (Woltz et al., 1992), suggesting a mechanism independent of pH. To understand the role of N in the management of FWB it is thus important to consider the effects of N fertilizer form and dose and the resulting change in pH.

Greater N availability has been proposed to cause a growth-defense trade-off in plants to maximize fitness (Herms and Mattson, 1992). Plants that grow more rapidly due to an abundance of N must sacrifice defensive capabilities due to greater metabolic expenditure, leaving them more susceptible to disease (Huot et al., 2014; Neuser et al., 2019). Pittaway et al. (1999) examined the relationship between FWB severity and pathogen invasion and activity under N-fertilized conditions. They hypothesized that the observed increase in disease severity was likely due to a predisposition of the host plant to disease, through a reduction in defense response, rather than changes in the pathogen. If the growth-defense trade-off is responsible for increased banana susceptibility to FWB in the future it may be possible to mitigate the effect through gene manipulation (Wang et al., 2021).

The diversity and biomass of soil bacterial and fungal communities, excluding the pathogen, generally are negatively correlated with the N fertilizer rate (Wang et al., 2018; Yang et al., 2020; Zhou et al., 2020). A reduction in microbial diversity and biomass with increased N fertilizer application is largely attributable to changes in soil pH (Wang et al., 2018; Yang et al., 2020). FWB is less severe in fields with high microbial diversity, possibly due to direct antagonism, resource competition, or support for the host plant defense (Shen et al., 2015; Fu et al., 2017; Rames et al., 2018; Zhou et al., 2019), so the application of N, which reduces microbial diversity of the soil, may increase disease severity. Therefore, it is important to consider both the rate of N applied and the form of fertilizer used.

The aim of this work was to determine the effects of inoculation with Foc, N rate and N form on the severity of FWB and the components of the FWB disease triangle. In controlled conditions, we examined the response of each component of the disease triangle: the pathogen (abundance in the rhizosphere by real-time qPCR), environment (rhizosphere soil chemistry and soil fungal and bacterial population diversity using ITS2 and 16S rRNA gene amplicon sequencing), and host (above- and below-ground growth, tissue N content and isotopic composition, and functional protein content of roots, using SWATH quantitative proteomics).

## Materials and methods

### Soil collection

The top 30 cm of a Liverpool series soil (Dermosol, Murtha, 1986) was collected from a commercial banana (*Musa* [AAA Group, Cavendish Subgroup] “Williams”) farm that had applied a maximum of 160 kg N/ha/year for the previous seven years. This location was selected to limit the presence of residual N fertilizer. Abiotic characterization of this soil (Site 5, Liverpool) has been published previously as part of a regional survey (Orr and Nelson, 2021). To prevent the spread of soil-borne disease, buckets of soil were sealed for transport and moved to the greenhouse where the experiment was conducted. Transport was at ambient temperature and took ~2 h. The soil was sieved to 10 mm to remove rocks, sticks, and large roots and then homogenized before potting.

### Greenhouse set-up

To each 4 L pot, 4 kg (dry weight equivalent) of soil was added. Foc Race 1-susceptible *in vitro* propagated banana plantlets (*Musa* [ABB Group, Pisang Awak Subgroup] “Ducasse”; Maroochy Research Facility, Maroochydore, Queensland) ~5 cm tall, were decanted and planted directly into soil. After 36 days, ammonium, as ammonium sulphate, or nitrate, as potassium and calcium nitrate, fertilizer were each applied at five rates (6.0, 12.1, 30.2, 48.3, and 66.4 mg N/pot/fortnight; equivalent to field rates of 50, 100, 250, 400, and 550 kg/ha/yr) to each pot. Potassium = 88.1, phosphorus = 6.64, calcium = 36.0, and sulphur = 84.1 mg/pot/fortnight were applied uniformly across the treatments, however the rate of chloride = 72.3 in ammonium pots and 57.0 mg/pot/fortnight in nitrate pots, and sodium differed between treatments (0–139 mg/pot/fortnight).

Fertilizers were applied in dissolved form by saturating the soil and then draining off the solution in excess of the soil's water holding capacity. This method allowed the experiment to be carried out in the soil while mimicking some of the benefits of hydroponic systems by redistributing solutes between rhizosphere and bulk soil (which deviated between fertilizer applications due to root activity), equilibrating soil water content between pots (which deviated between pots due to differential transpiration) and removing nitrate (generated by nitrification) from ammonium-treated pots. The water holding capacity of each pot was determined by saturating the known dry weight of soil in each pot with water and then allowing it to drain freely for 24 h and was no longer dripping before reweighing. Prior to each fertilizer application, each pot was watered to 200 ml below water holding capacity by weight. Then the pot was soaked in a fertilizer solution with the correct amount of fertilizer per 200 ml. The pot contents would absorb 200 ml of solution, through small holes at the bottom of the pot over a period of ~5 min, bringing the pot contents to their water holding capacity and required fertilization. Then the pot was removed from the fertilizer solution and allowed to freely drain for 24 h before weighing. The difference between the weight prior to immersion, and that after freely draining was used to confirm the amount of fertilizer retained. The pot weight after free draining was also used to calculate the water required prior to fertilizer addition for the next fertilizer addition 14 days later.

After 61 days, 7.5 ml of millet inoculated with a mixture of two Foc isolates- (Race 1, VCG 0124 [BRIP 43996 and BRIP61873]; Queensland Plant Pathology Herbarium) was added to the inoculated treatment pots. Inoculated millet was prepared following the method of Warman and Aitken (2018) except the millet was not ground prior to inoculation of the pots. Autoclave-sterilized millet was added to the remaining pots, resulting in 20 unique treatment combinations. Inoculated treatments were replicated 5 times, to account for the greater variability of incorporating an extra treatment, and non-inoculated 4 times (*n* = 90). The millet was mixed into the top 20 mm of soil in each pot, ensuring not to cross contaminate the treatments. All other soil conditions, including temperature and water supply, were consistent between treatments.

### Sampling

Soil samples for pH, nitrate, and ammonium analysis were collected 1, 36, and 52 days after inoculation. Samples for root proteome and rhizosphere soil qPCR and amplicon sequencing were collected 52 and 53 days after inoculation by gently removing the plant and roots from the pot and taking the sample before replanting the plant and remaining roots in the pot. These samples were taken prior to plant harvest to ensure the plant stress response was not due to harvest. Plants were harvested over a 2-day period, 56 and 57 days after inoculation, after severe leaf yellowing and pseudostem splitting had been observed in some plants.

At harvest, the roots were separated from the rhizome and kept separate to minimize soil contamination. The rhizome was sectioned for the determination of disease severity (see below). The above-ground (including rhizome) and root sections were weighed for fresh weight, dried at 60°C until a constant dry weight was achieved, and reweighed to determine the dry weight and water content of the tissue. The entire dried above-ground section of the plant was then ground to a fine powder and homogenized prior to analysis of total N and carbon (C) concentration, and stable isotope composition.

### Disease severity

Internal disease severity was determined using the method of Orr et al. (2019). Briefly, after the removal of the roots, the rhizome was laterally sectioned into quarters and the upper side of each section was photographed. Each pixel of the section photographs was classified as either diseased or not diseased using the image analysis program ImageJ v1.52a (Schneider et al., 2012) and a reference grayscale. For sections where the upper side of the lateral section mistakenly included pseudostem tissue rather than the rhizome, a value was not recorded. The proportion of the total rhizome pixels classified as diseased was used as a percentage of disease severity for each rhizome section. The values for each rhizome section were then averaged to provide a disease severity value for that plant.

### Soil pH, nitrate, and ammonium analysis

Soil samples were collected 1, 36, and 52 days after inoculation. A 10 mm internal diameter, 300 mm length, stainless steel tube was inserted to the complete depth of the pot to collect a core. The tube was cleaned and sterilized between samples to avoid cross-contamination. Each core was homogenized and 4 g (dry weight equivalent) of the sample was end-over-end shaken for 1 h with 40 ml of 0.1 M potassium chloride before centrifuging at 3,000 × *g* for 5 min. After centrifuging the supernatant pH was measured (Ionode IH series probe; Ionode Pty Ltd. Australia) and subsamples of the supernatant were taken for nitrate and ammonium analysis.

Nitrate analysis followed Hach method 8171 (Hach, 2017), using a linear six-point calibration curve from analytical grade standards (Hach, Australia). Ammonium was determined using the Hach method 8038 (Hach, 2017), also using a linear six-point calibration curve from analytical grade standards (Hach Australia).

### Plant N and C concentration, and isotopic composition

Total N concentration of the plant (aboveground portion) was measured to determine N uptake, and δ^15^N was measured to determine if the treatments influenced the proportion of N taken up directly from fertilizer vs. soil or microbially processed N (Denk et al., 2017). The δ^13^C of the same samples was measured to determine effects on water use efficiency (Cernusak et al., 2013). C and N concentrations, and δ^13^C and δ^15^N values of the plant, soil and fertilizer samples were determined using a Costech Elemental Analyzer fitted with a zero-blank auto-sampler coupled *via* a ConFloIV to a ThermoFinnigan DeltaVPLUS using Continuous-Flow Isotope Ratio Mass Spectrometry (EA-IRMS) at James Cook University's Advanced Analytical Centre (Cairns). Stable isotope results are reported as per mille (‰) deviations from the VPDB and AIR reference standard scale for δ^13^C and δ^15^N values, respectively. The standard deviation on internal standards was <0.1 and 0.2% for C and N, respectively. As δ^15^N differed between the fertilizers used (ammonium sulfate, sodium nitrate, and potassium nitrate), the treatment effect on the isotopic composition of plant material was calculated using δ^15^N_Plant_–δ^15^N_Fert_.

### Amplicon sequencing and qPCR

#### Sample collection and extraction

Rhizospheric soil was collected based on previously published methods (Birt et al., 2021, 2022). Briefly, ~70 mm long sections from the apex of three white roots of each plant, with rhizosphere soil attached, were cut with methanol-sterilized scissors and stored at 4°C until processing on the same day. Samples were sonicated in 40 ml phosphate-buffered saline solution to loosen soil and fine roots. Large roots were then removed and the remaining soil suspension was centrifuged for 15 min at 400 × *g* before removal of the supernatant. The remaining soil pellet (representing the rhizosphere) was frozen at −18°C until DNA extraction the following week.

DNA was extracted using a Qiagen DNeasy Powersoil kit (Qiagen, Australia) according to manufacturer recommendations. The amount of soil extracted for each sample was weighed precisely for use in calculating DNA concentrations in soil. The extracted rhizosphere soil DNA sample was then used for both Foc R1 qPCR and amplicon sequencing.

#### PCR amplification and sequencing

To survey bacterial communities, 16S rRNA genes were amplified by PCR using the primers 926wF (5′-AAA CTY AAA KGA ATT GRC GG-3′) and 1392R (5′- ACG GGC GGT GWG TRC−3′) (Lane, 1991). Fungal communities were surveyed using the ITS2 rRNA gene primers gITS7 (5′- GTG AAT CAT CGA ATC TTT G-′3) (Ihrmark et al., 2012) and ITS4 (5′- TCC TCC GCT TAT TGA TAT GC-′3) (White et al., 1990). Thermocycling conditions were as follows for all reactions: 98°C for 45 s; then 35 cycles of 98°C for 5 s, 56°C for 5 s, 72°C for 6 s; followed by 72°C for 1 min. Each PCR reaction contained 2 μL DNA of the sample in 5X Phire Green Reaction Buffer (Thermo Fisher), 0.4 μl Phire Green Hot Start II DNA Polymerase (Thermo Fisher), 100 μM of each of the dNTPs (Invitrogen), and 10 mM of each primer. The remaining volume was made to 20 μl with molecular-grade water. A Simpliamp^®^ 96-well Thermocycler (Applied Biosystems) was used to perform PCR reactions. Gel electrophoresis was used to verify blank extraction controls and negative amplification controls.

Magnetic beads (Rohland and Reich, 2012) were used to purify amplicons, which were then dual indexed using the Nextera XT Index Kit (Illumina) according to the manufacturer's instructions. Indexed amplicons were again purified using magnetic beads and quantified using a PicoGreen dsDNA Quantification Kit (Invitrogen). Samples were pooled in equal concentrations and sequenced on an Illumina MiSeq using 30% PhiX Control v3 (Illumina) and a MiSeq Reagent Kit v3 (600 cycles, Illumina) according to the manufacturer's instructions.

Sequence data were processed using a modified UPARSE approach (Edgar, 2013). Firstly, reads were demultiplexed and barcodes removed using the cutadapt tool in QIIME2 (v2017.9.0; Zhang et al., 2000. ITS2 or 16S sequences were then each processed according to a different protocol. For 16S sequences, primers were then removed and trimmed to 250 bp using fastx_truncate of USEARCH (Edgar, 2010). Trimmed reads were then quality filtered using fastq_filter of USEARCH with a maxee score of 1.0. For ITS2 sequences, ITSx (Bengtsson-Palme et al., 2013) was used to extract the ITS2 region with fungi as the specified profile. Chimeras were then removed from the extracted sequences using uchime2_ref of USEARCH against the UNITE 8.2 database (Nilsson et al., 2019). ITS2 and 16S sequences then resumed with the same pipeline after these steps had been completed. Representative sequences were generated using fastx_uniques and cluster_otus of USEARCH with a sequence similarity of 0.97. These representative sequences were used to create an operational taxonomic unit (OTU) table by mapping the remaining reads using the otutab function in USEARCH. Taxonomy was assigned to each OTU using blastn from QIIME2 against the SILVA 128 (Quast et al., 2013) database for 16S sequences and UNITE 8.2 database for ITS2 sequences. Sequences that were not either bacteria or fungi were then removed using taxa filter-table from QIIME2. The 16S sequences were then aligned using MAFFT (Katoh and Standley, 2013) and masked using QIIME2 to generate phylogenetic distance. A midpoint-rooted phylogenetic tree was generated from the alignment using FastTree (v2.1.9) (Price et al., 2010). 16S samples were rarefied to 6950 reads, ITS samples were rarefied to 13,500. Alpha diversity metrics were produced using QIIME2.

#### qPCR analysis

Foc abundance was quantified with absolute quantification-based qPCR as previously published (Matthews et al., 2020), except for the use of SsoAdvanced SYBR^®^ SuperMix (BioRad), which has been shown to increase reaction efficiency compared to other qPCR master mixes (Browne et al., 2020). Briefly, Foc-specific DNA sequences were targeted using the forward priming sequence (5′-GACATTTGACGACTTTCTGA-3′), the reverse sequence (5′-GACATTTGACGACTTTCTGA-3′) (Matthews et al., 2020). Each 10 μl total-volume reaction contained 5 ng of extracted DNA, 0.3 μM of desalt-grade primers (Sigma-Aldrich), 5 μl of SsoAdvanced SYBR^®^ SuperMix, and 2 μl molecular grade Ultra-Pure H_2_O™ (Invitrogen) water. Samples were amplified in a reaction including an initial hot start of 10 min at 95°C, followed by 40 cycles of 10 s at 95°C and 15 s at 66°C. Reactions were followed by a melt curve analysis to ensure primer specificity. Furthermore, technical triplicates were run alongside no template negative controls as well as a sample, extraction, and analysis blank. The entire dilution and technical triplet analysis were also run 2 times to check dilutions. Values from the two dilution sets were consistent and values were averaged. The reaction amplification efficiency was calculated from the gradient of the standard curve titration in accordance with MIQE guidelines (Bustin et al., 2009). Cycle threshold (Ct) scores were converted to DNA copy numbers using a standard curve constructed from a pGEM-T plasmid carrying the Foc specific DNA directed RNA polymerase subunit III gene (*i.e*. 10^8^−10^1^ copies plasmid/reaction). The target amplicon was 98 base pairs long, reaction efficiency was 98.1%, and standard curve R^2^ was 0.99. Reactions were measured by QuantStudio 5 Real-Time PCR Machine running QuantStudio Design and Analysis Software (v1.4.3, Applied Biosystems).

### Plant proteomics

#### Sample collection and extraction

When sampling each plant, three healthy root ends (~5 cm long) were cleaned with deionized water to remove soil while attached to the plant, cut, immersed in liquid N, and ground to a fine powder in a liquid-N-cooled mortar and pestle. Samples were then stored at −80°C until processing. Between samples, all surfaces were thoroughly cleaned with methanol to prevent cross-contamination. Peptides were extracted using an acid digestion method adapted from the work of Doellinger et al. (2020). For the full method used, see Supplementary Information 1.

#### LC-MS/MS analysis

All samples were analyzed with a Ekspert nano-LC415 (Eksigent, USA) liquid chromatography (LC) system running on the water with 0.1% (v/v) formic acid in water (Solvent A) and 0.1% (v/v) formic acid in Acetonitrile (Solvent B), coupled to a TripleTOF 6600 (Sciex, USA) mass spectrometer (MS). Digested peptides were first loaded on a C18 10 mm by 300 μm trap column (Trajan, Australia) under 10 μl/min of Solvent A and separated on a C18 250 mm by 300 μm column (Trajan, Australia) with a linear gradient of 3–35% solvent B over 75 min at 5 μl/min. Mass spectrometer experimental parameters (Sciex software units) were as follows: curtain gas = 35, ion source gas 1 = 25, ion source gas 2 = 30, ionspray voltage floating = 500, and turboheater temperature = 300°C.

A 0.2 μl aliquot of a replicate from each treatment was pooled into two samples and loaded into the LC-MS system. Information Dependent Acquisition mode was set to capture ions with a charge state between 2 and 5 and at a window of 350–750 Da and 745–1,250 Da respectively. A single spectral library was generated from the MS data from both windows for all conditions with the ProteinPilot (Sciex, USA) Paragon method identification workflow under the following settings: Cysteine Alkylation = Iodoacetamide w other Cys mods possible, Digestion = Trypsin, Instrument = TripleTOF 6600, Search effort = Through, ID Focus = Biological modifications, and Unused ProtScore = 0.05. The MS data was matched against a *Musa acuminata* subsp. *malaccensis* protein library containing 45,856 protein sequences, obtained from the Banana genome hub in January 2020 (Droc et al., 2013).

The same physical set-up was used in “Sequential Windowed Acquisition of All Theoretical Fragment Ion Mass Spectra” (SWATH) mode to acquire quantitative data for all 90 samples. The SWATH method used 100 windows with 6-50 Da of isolation width, an overlap of 1 Da, and a collision energy spread of 5-10 through the mass range of 399.5 Da to 1249.5 Da. Data was collected over 1,847 cycles with an accumulation time of 25 ms for each window.

SWATH data was processed using the SWATH microapp on the PeakView software (Sciex, USA). The retention time for all SWATH samples was normalized with five selected peptides from the iRT calibrant (Biognosys, Switzerland) ranging from 487.26 to 699.34 *m/z*. All samples were processed with the previously generated spectral library according to the following SWATH microapp settings: number of peptides per protein = 9, number of transitions per peptide = 9, peptide confidence threshold = 99%, false discovery rate threshold = 1%, XIC extraction window = 20 mins, and XIC width = 50 ppm.

### Statistical analyses

#### Univariate analyses

Unless otherwise specified, data were analyzed using an ANCOVA with the ANOVA function in the *car* package in R software (R Core Team, 2017; Fox and Weisberg, 2019). This method was selected to address the unbalanced study design with respect to inoculation, incorporating type 3 sum of squares error. The predictors included N rate, considered as a continuous variable, N form, a factor with two levels (ammonium and nitrate), and sometimes inoculation when considering both inoculated and non-inoculated plants. The normality of the model residuals was tested using the Shapiro Wilks test and data was transformed if needed. Soil nitrate concentration and fungal Chao1 index were log_e_(x+1) transformed, plant N tissue concentration and rhizosphere Foc DNA concentration were square root transformed, and the fungal Shannon index was cubed. Soil ammonium concentration and bacterial and fungal Simpson indexes were not normally distributed even when transformed so they were analyzed using non-parametric methods with the *ARTool* package (Kay et al., 2021) with N rate and form considered as factors. Untransformed data are presented in figures for ease of interpretation. For determining a significant effect in the tested models α = 0.05 was used, unless otherwise specified.

The effects of the treatments on disease severity were analyzed using a beta regression analysis with a logit link in R using the *betareg* package (Cribari-Neto and Zeileis, 2010). This was selected due to disease severity being a percentage, with a maximum of 100%. Effects on inoculated plant dry weight were analyzed using an ANOVA, but a second order polynomial function was incorporated for the N rate. For both disease severity and inoculated plant weight, a single outlier value was excluded as it was three standard deviations from the mean. The replicate was not an outlier in other analyses possibly indicating an analytical error.

#### Multivariate analyses

To calculate the treatment effects on microbial beta diversity a PERMANOVA analysis was performed using the adonis function in the *vegan* R package (Oksanen et al., 2019). For determining a significant effect in the tested models α = 0.05 was used, unless otherwise specified.

Proteomic data were quantile normalized and log_2_ (x+1) transformed for analysis (Ritchie et al., 2015). PERMANOVA was used to determine the significance of treatments and soil pH on protein expression composition. Redundancy analysis was performed using the *vegan* package in R (Oksanen et al., 2019), constrained by the treatments of N rate and inoculation as these were found to be significant. Differentially expressed proteins were identified and enrichment was calculated in the *limma* package in R with a false discovery rate cut-off of 0.10 incorporating a Benjamini–Hochberg correction for multiple testing (Ritchie et al., 2015; Law et al., 2020) using a full model incorporating the treatments of inoculation, N rate and form as well as all interactions. Pathway enrichment was then explored on each main or interaction treatment protein set. Further testing was performed on the expression rates of key individual defensive genes using a full treatment model. Due to concerns regarding the assumption of linearity for protein expression changes, N was also considered as a multilevel factor; however, the results were simply a subset of the linear regression analysis due to the reduction in statistical power so were not reported here.

Gene ontology enrichment was calculated using the *topGO* package in R (Alexa and Rahnenfuhrer, 2020). Protein IDs were mapped to gene ontology using the Banana Genome Hub (Droc et al., 2013). Fisher's exact test was used to determine enrichment using conservative cut offs (α = 0.01 and >3 proteins per ontology). KEGG pathway enrichment was calculated by annotating the full list of inferred genes analyzed in this experiment using the FASTA sequence blast function in BlastKoala as part of the KEGG mapping service (Kanehisa et al., 2016). KEGG k values from the full experimental protein set were mapped to pathway ko numbers to indicate the protein universe within the samples. Pathway enrichment was determined using a hypergeometric test for each KEGG pathway. This test compared the number of k values (indicating individual proteins or genes) in the differentially expressed protein set with the number of k values in the protein universe. Here, the protein universe was limited to proteins analyzed in this experiment, rather than the entire banana proteome (Bessarabova et al., 2012). Cut offs for enrichment were the same as for gene ontology.

## Results

### Plant growth and disease severity

**Figure 1:**
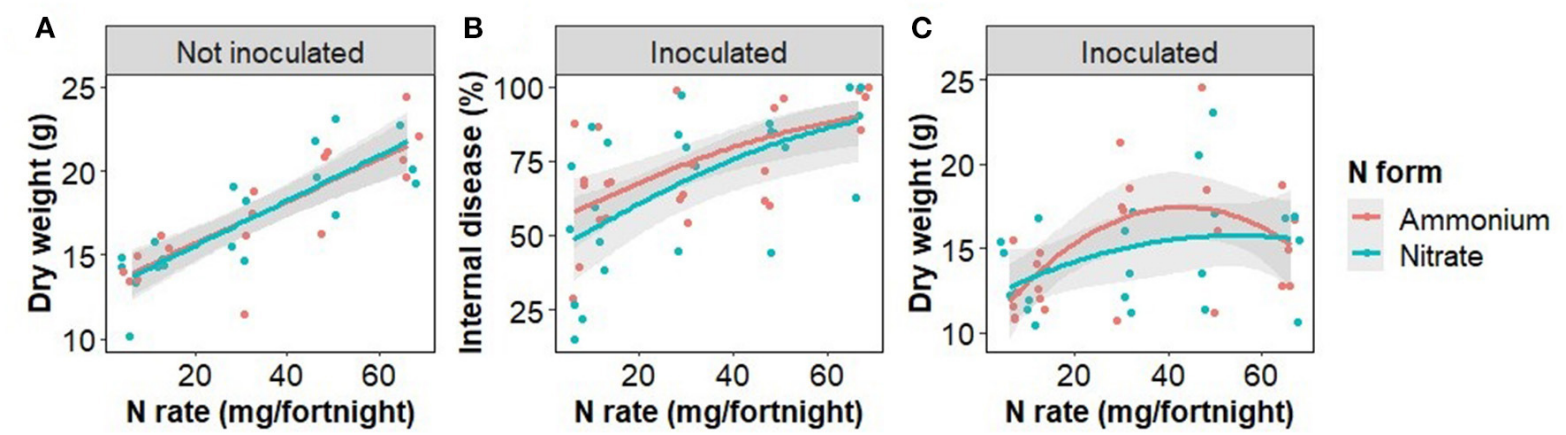
The effect of nitrogen fertilizer rate on total plant dry weight in plants that were not inoculated with *Fusarium oxysporum* f.sp. *cubense* **(A)**, disease severity in inoculated plants **(B)** and dry weight in inoculated plants **(C)**. For regression model statistics see Table 1. Points have been jittered in the x dimension to avoid overplotting.

The rate of N fertilizer application significantly affected plant biomass as well as FWB severity (Figure 1; Table 1). Plant dry weight in non-inoculated plants responded linearly to the N rate (Figure 1). Disease severity was significantly related to the N rate in inoculated plants, the rate of increase being constrained by the maximal (100%) internal disease. The combined effects on plant growth and disease severity resulted in a second order polynomial relationship between N rate and plant weight in inoculated plants (Figure 1).

**Table 1:** The model outputs for the relationship between plant weight and treatments for plants inoculated with *Fusarium oxysporum* f.sp. *cubense* (quadratic) and not inoculated (linear) as shown in Figure 1.

**Response variable**	**Predictor variable**	**Inoculated**		**Not inoculated**	
Plant weight		*F*	*p*	*F*	*p*
	Intercept	35.4	<0.001	259.4	<0.001
	N rate	5.5	0.008	37.1	<0.001
	N form	0.7	0.394	0.3	0.610
	N rate ^*^ N form	0.7	0.483	0.8	0.378
Disease severity		*z_43_*	*p*		
	Intercept	0.4	0.676		
	N rate	3.8	<0.001		
	N form	−1.1	0.286		
	N rate ^*^ N form	0.6	0.520		

The * symbol signifies the interaction. An alternative to the symbol would be ‘x'.

### Soil chemistry

**Figure 2:**
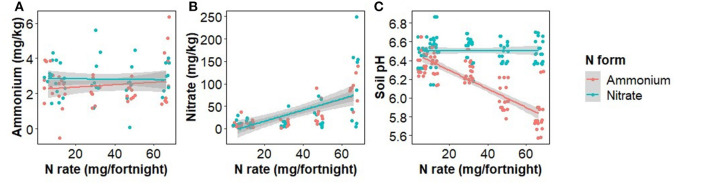
The effect of ammonium and nitrate fertilizer addition on the concentration of soil ammonium **(A)**, nitrate **(B)**, and pH **(C)** at the time of harvest.

The concentration of ammonium in soil was unaffected by the rate, form, or interaction of rate and form of N fertilizer used, whereas the concentration of nitrate in soil was positively correlated with the N application rate (*F*_(1,86)_ = 44.9, *p* < 0.001), but not the form or the interaction of rate and form (Figure 2). Soil pH was significantly affected by the interaction of rate and form (*F*_(1,86)_ = 51.4, *p* < 0.001) with ammonium addition decreasing soil pH (Figure 2).

### Plant N uptake and water use efficiency

**Figure 3:**
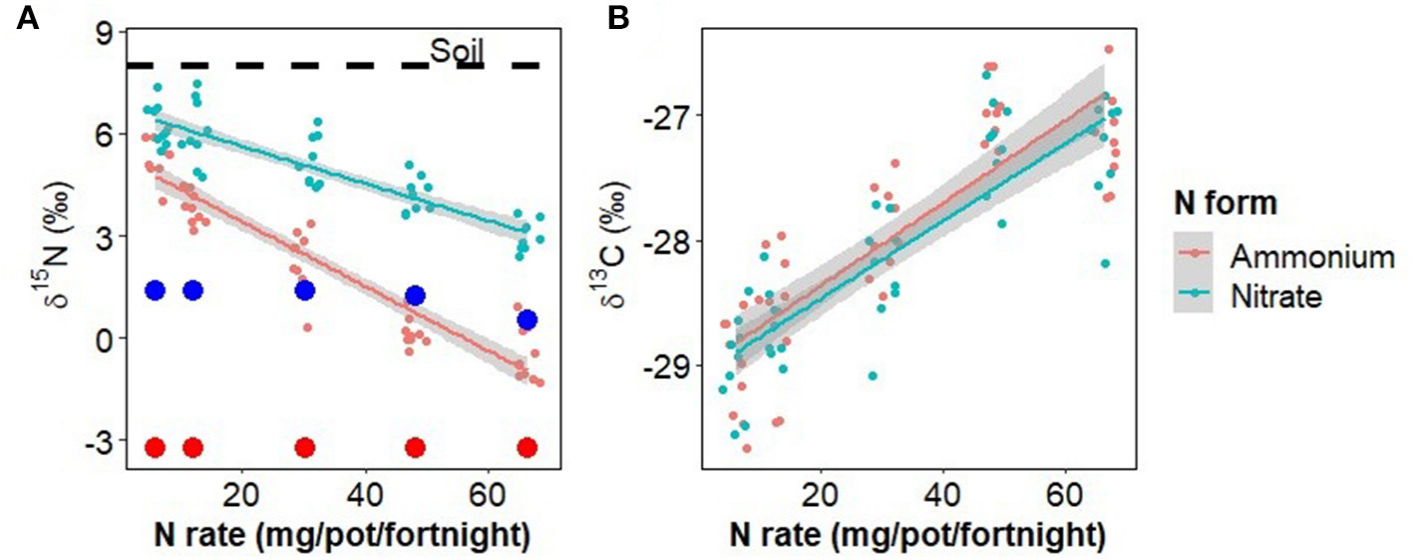
**(A)** The effect of nitrate and ammonium fertilizer addition on the δ^15^N of aboveground plant tissue, showing values for the original soil (dotted line) and fertilizer (large colored points for each fertilizer type and rate, red = ammonium, blue = nitrate). **(B)** δ^13^C of aboveground plant tissue. Points have been jittered in the x dimension to avoid overplotting.

The N concentration of aboveground plant tissue increased significantly with N fertilizer application rate [0.87−3.28 %, *F*_(1,82)_ = 128.7, *p* < 0.001], but was not significantly affected by N fertilizer form, inoculation, or any interactions. The difference between δ^15^N_Plant_ and δ^15^N_Fert_ was significantly affected by N rate [*F*_(1,82)_ = 228.7, *p* < 0.001], N form [*F*_(1,82)_ = 100.2, *p* < 0.001], and the interaction of N rate and form [Figure 3, *F*_(1,82)_ = 31.7, *p* < 0.001], but not inoculation or any other treatment combinations. When fertilizer rates were low, δ^15^N_Plant_ values were similar to that of unfertilized soil, but as fertilizer rates increased δ^15^N_Plant_ decreased, approaching that of the fertilizer (difference of zero). δ^13^C was significantly increased by N rate [Figure 3, *F*_(1,82)_ = 62.7, *p* < 0.001], but no other treatments or combinations.

### Foc abundance

**Figure 4:**
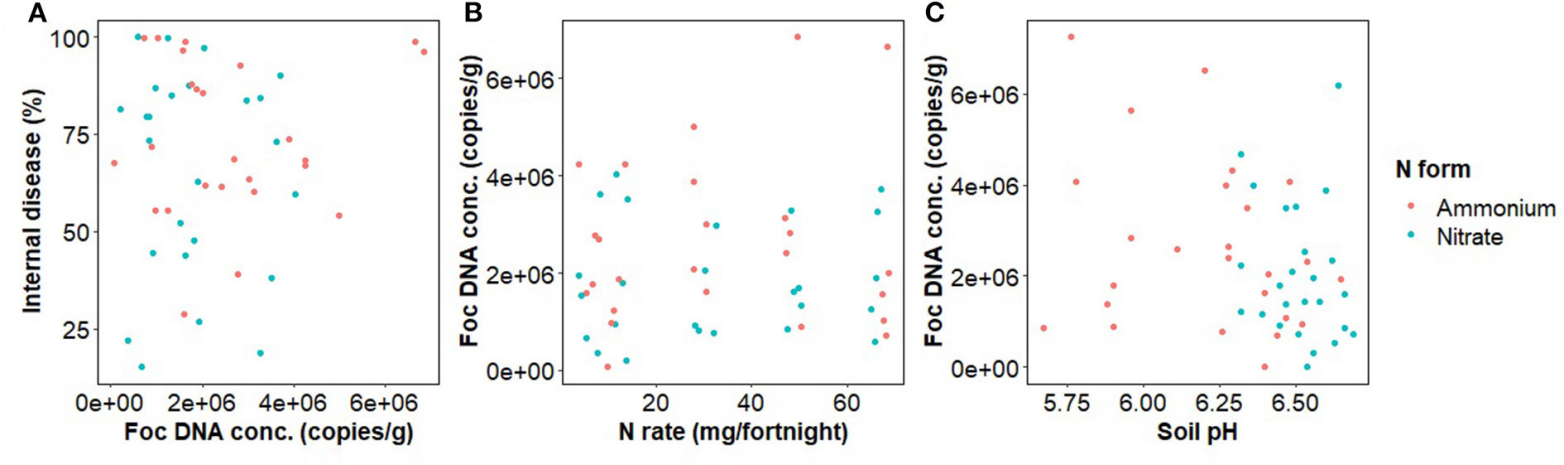
*Fusarium oxysporum* f.sp. *cubense* DNA concentration in banana rhizosphere soil in relation to nitrogen fertilizer form and **(A)** Internal disease severity of banana plants, **(B)** Nitrogen fertilizer rate, and **(C)** Soil pH.

The concentration of Foc DNA in rhizosphere soil of inoculated pots had no relationship with banana internal disease severity (Figure 4). Foc DNA concentration was not significantly affected by N rate, form, or their interaction but was marginally negatively related to soil pH [*F*_(1,48)_ = 2.8, *p* = 0.098] (Figure 4).

### Soil bacterial and fungal diversity

**Figure 5:**
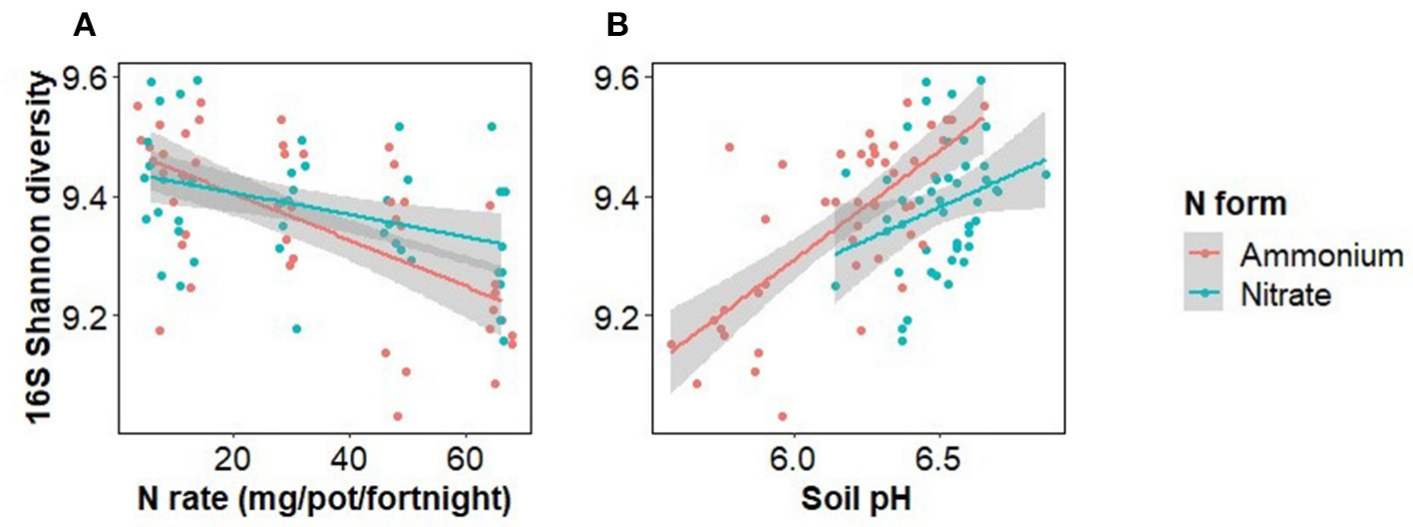
Bacterial alpha diversity (Shannon index) as affected by the rate and form of nitrogen fertiliser addition **(A)** and soil pH **(B)**. Points in the left panel have been jittered in the x dimension to minimize overplotting.

Bacterial alpha diversity was significantly decreased by N rate, but not significantly affected by N form or inoculation (Sobs, Chao1, Shannon, Simpson, and PD indices) (Figure 5, Supplementary Information 2). An ANOVA with N form and either soil pH or N rate was run to test the relative influence of soil pH and N rate on Shannon diversity, as well as the importance of the interaction of these terms. Both soil pH and N rate were significant predictors of Shannon diversity but soil pH was stronger. In addition, there was a significant interaction between N rate and N form (F = 4.3, *p* = 0.042) but not between soil pH and N form (F = 1.4, *p* = 0.234). Bacterial beta diversity, as tested with a PERMANOVA, was significantly affected by the main effects of inoculation (F = 1.5, R^2^ = 0.017, *p* < 0.001), N rate (F = 2.2, R^2^ = 0.024, *p* < 0.001) and form (F = 1.9, R^2^ = 0.020, *p* < 0.001), as well as the interactions of N rate and form (F = 1.7, R^2^ = 0.018, *p* < 0.001) and N rate and inoculation (F = 1.2, R^2^ = 0.012, *p* = 0.042). Soil pH and N form both significantly affected bacterial beta diversity but their interaction was not significant (F = 1.1, R^2^ = 0.012, *p* = 0.265), whereas when N rate and N form were considered, the interaction was significant (F = 1.7, R^2^ = 0.018, *p* < 0.001).

**Figure 6:**
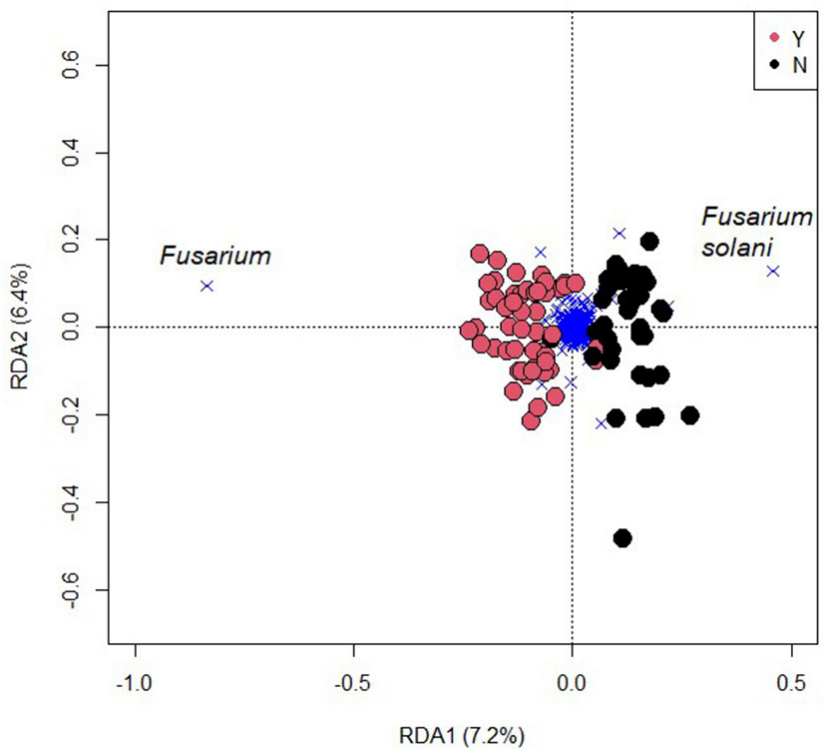
A redundancy plot of the fungal community with inoculation of *Fusarium oxysporum* f.sp. *cubense* (Y) or not (N) constrained by inoculation. Circles represent samples and crosses OTUs. The far-left cross represents the genus *Fusarium*, which exerts considerable influence on the significance of the treatment effect.

Fungal beta diversity was affected by inoculation (F = 6.8, R^2^ = 0.072, *p* < 0.001; Figure 6), but was not significantly affected by N rate, N form, or any interactions. The species *F. solani* was most representative of uninoculated samples while the fungal OTU most affected by inoculation was the genus *Fusarium* (Figure 6). Fungal alpha diversity measures that considered only richness (Chao1 and Sobs) were not affected by inoculation; however, those that also considered evenness (Shannon and Simpson) were indicating that some OTUs were reduced in abundance but not removed in entirety (Supplementary Information 2).

### Plant proteomics

**Figure 7:**
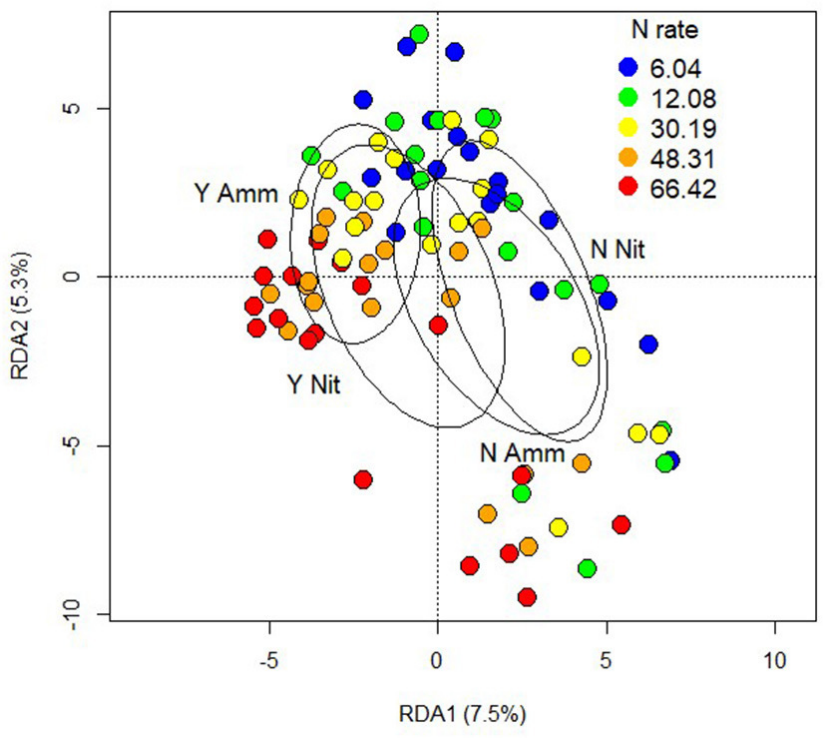
Redundancy analysis of the proteomic output constrained by factors of inoculation, nitrogen form and nitrogen rate. Point colours indicates nitrogen rate and ellipses with text labels indicate combinations of inoculation (Y or N) with nitrogen form (Ammonium or Nitrate).

A full factorial PERMANOVA analysis of the proteome dataset considering inoculation, N rate and form demonstrated a large effect of inoculation (F = 6.0, R^2^ = 0.059, *p* < 0.001), and N rate (F = 6.0, R^2^ = 0.060, *p* < 0.001) and the interaction of all three treatments was significant (F = 2.0, R^2^ = 0.019, *p* < 0.027). N form and the other interactions were not significant (Figure 7). When soil pH was included in the model it was not significant, nor were any interactions with it.

Examination of differential expression on the individual protein level showed that the main effects of N rate and inoculation had the greatest effect, but there was no main effect of N form. N form and inoculation had the sole interaction with differential expression though none of the identified proteins were related to growth or defense. Therefore, further investigation was focused on those proteins differentially expressed under the main effects of N rate and inoculation. The false discovery rate used for differential expression was highly conservative due to the large number of tests performed and the multiple testing adjustments required. A separate investigation of key defensive proteins included all treatments and their interactions.

Inoculation with Foc resulted in differential expression of the proteins associated with the “Defense response” process as well as a wide range of other molecular functions (Supplementary Information 3). KEGG pathway analysis identified differentially expressed groups of proteins primarily related to the metabolism of sugars (Supplementary Information 3).

Gene ontology and KEGG pathway enrichments affected by N rate were primarily processes and functions associated with energy fixation and use (Tables 2, 3). The KEGG MAPK signaling pathway was also affected, indicating that the addition of N may have altered the plants' ability to respond to biotic and abiotic stressors (Table 3).

**Table 2:** Significantly differentially expressed gene ontologies based on nitrogen rate.

**GO ID**	**Term**	**Annotated**	**DE**	***P***	**Sub ontology**
GO:0033897	Ribonuclease T2 activity	5	3	0.006	MF
GO:0008270	Zinc ion binding	18	5	0.018	MF
GO:0008964	Phosphoenolpyruvate carboxylase activity	3	2	0.023	MF
GO:0003735	Structural constituent of ribosome	111	16	0.035	MF
GO:0005471	ATP:ADP antiporter activity	4	2	0.043	MF
GO:0004448	Isocitrate dehydrogenase activity	4	2	0.043	MF
GO:0004634	Phosphopyruvate hydratase activity	4	2	0.043	MF
GO:0015977	Carbon fixation	3	2	0.024	BP
GO:0006412	Translation	152	19	0.036	BP
GO:0006099	Tricarboxylic acid cycle	15	4	0.042	BP
GO:0005840	Ribosome	112	16	0.020	CC
GO:0016272	Prefoldin complex	7	3	0.023	CC
GO:0000015	Phosphopyruvate hydratase complex	4	2	0.049	CC

**Table 3:** Significantly differently expressed KEGG pathways, based on proteins that were differentially related to nitrogen rate.

**K**	**Function**	**Measured**	**DE**	***P***
00710	Carbon fixation in photosynthetic organisms	16	6	0.003
03018	RNA degradation	8	3	0.016
04016	MAPK signalling pathway—plant	9	3	0.026
03010	Ribosome	71	15	0.030
00270	Cysteine and methionine metabolism	19	5	0.038
00230	Purine metabolism	10	3	0.039

**Figure 8:**
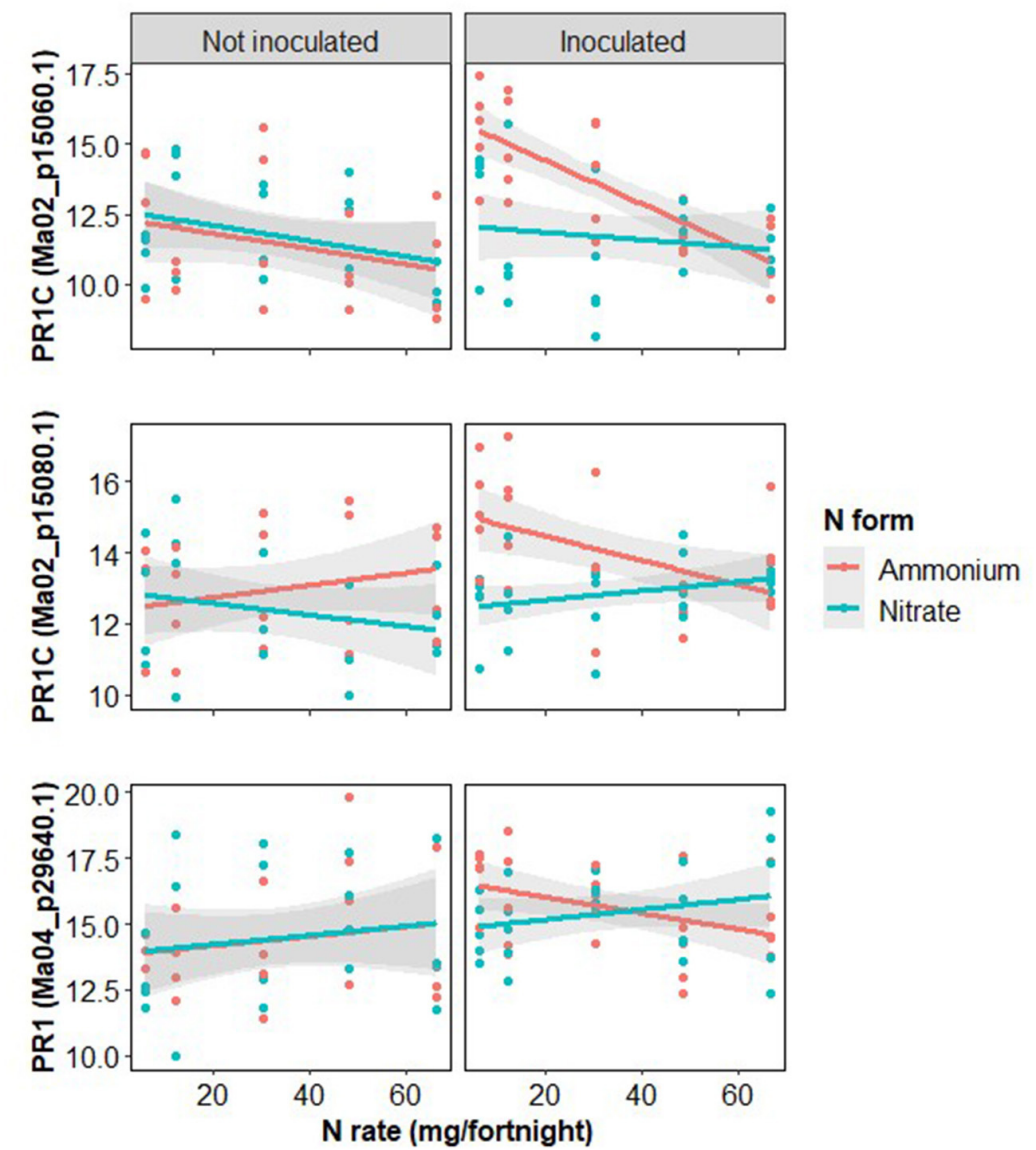
Log_2_ transformed expression rates of measured forms of pathogenesis related protein 1 (PR1), shown with gene locations, with treatments of inoculation with *Fusarium oxysporum* f.sp. *cubense*, nitrogen rate and form.

Pathogenesis-related protein 1 (PR1) expression rates were examined due to their importance as late-stage biotic infection response proteins. Inoculation significantly increased the expression of all three PR1 proteins (Table 4; Figure 8). All three forms of PR1 showed strong and complex interactions between the three treatments so the N rate response was examined separately for each combination of inoculation and N form. Increased N rate significantly reduced PR1 expression in inoculated plants treated with ammonium but there was not a significant effect of N rate on inoculated plants treated with nitrate, nor on disease-free plants fertilized with either form of N (Figure 8).

**Table 4:** Effects of inoculation, nitrogen (N) rate and N form on the three forms of Pathogenesis related protein 1 measured.

**Predictor variable**	**Ma02_p15060.1**		**Ma02_p15080.1**		**Ma04_p29640.1**	
	***F**_(1,82)_*	***p***	***F**_(1,82)_*	***p***	***F**_(1,82)_*	***p***
N rate	2.3	0.133	1.5	0.227	0.8	0.377
N form	0.1	0.788	0.4	0.524	0.0	0.967
Inoculation	13.6	<0.001	13.3	<0.001	6.9	0.01
N rate ^*^ N form	0.0	0.994	2.8	0.098	0.0	0.995
N rate ^*^ Inoculation	4.1	0.047	7.3	0.008	3.2	0.076
N form ^*^ Inoculation	9.0	0.004	9.2	0.003	1.6	0.214
3 way interaction	3.4	0.068	9.0	0.004	1.7	0.193

The * symbol signifies the interaction. An alternative to the symbol would be ‘x'.

## Discussion

Disease severity, determined here by the proportion of plant tissue that was necrotic, was examined in response to Foc infection, N fertilizer rate and form, and the interaction of these treatments. In Foc inoculated plants, disease severity was positively correlated with N fertilizer rate in either N form irrespective of changes to soil pH, resulting in curvilinear growth response to increased N (Figure 1). This result agrees with previous findings that N is positively correlated with disease severity of both Foc Race 1 and Tropical Race 4 (Segura-Mena et al., 2021; Teixeira et al., 2021), though disagrees with the general trend that acidification associated with the ammonium use increases Fusarium wilt and FWB severity, whereas nitrate use is protective (Orr and Nelson, 2018). δ^13^C was also positively associated with the N rate in both inoculated and disease-free plants indicating that water use efficiency of photosynthesis, of which δ^13^C is a proxy (Farquhar et al., 1982), was affected by N availability as expected (Evans, 1989) but not by the presence of water restricting wilt symptoms.

The disease is the result of the interactions between the three components of the disease triangle: plant, pathogen, and environmental influences (Agrios, 2005). We found that soil Foc load was a poor predictor of disease severity and only marginally related to N rate or soil pH, in agreement with Pittaway et al. (1999). Soil bacteria and fungi populations were more consistently affected by soil pH and the introduction of the pathogen than by N rate *per se* as the N rate effect differed between N forms (Figure 4; Supplementary Information 2). High rates of N fertilizer, and associated increased plant growth, are often linked with increases in disease due to a trade-off between growth and defense (Herms and Mattson, 1992; Huot et al., 2014). The expression of proteins associated with the “MAPK signaling pathway—plant,” plant stress and defense pathway, was significantly changed by increased N supply, independent of the presence of the pathogen. PR1 expression, a key endpoint for the “MAPK signaling pathway—plant” and salicylic acid defense response was negatively affected by ammonium fertilization in diseased plants. N fertilization enriched the “Carbon fixation in photosynthetic organisms” pathway that is a key contributor to growth. This change was likely associated with an increase in photosynthetic efficiency shown by the measured enrichment in δ^13^C with N fertilization. Taken together, these findings support the growth-defense trade-off hypothesis, which suggests that an increase in disease severity results from a reduction in host plant defense resulting from an increase in growth, and a pH effect on the soil microbial community.

Auxin-induced plant growth, triggered by the addition of N, suppresses defensive processes such as salicylic and jasmonic acid signaling (Yaeno and Iba, 2008; Guo et al., 2018; Van Butselaar and Van Den Ackerveken, 2020). Jasmonic acid production is the primary response to necrotrophic pathogens, whereas salicylic acid production is the primary response to biotrophic and hemibiotrophic pathogens such as *F. oxysporum* f.sp. *cubense*, though there is important crosstalk between the two responses (Di et al., 2016; Sun et al., 2020; Fernandes and Ghag, 2022). The induction of auxin and target of rapamycin pathways, which are associated with plant growth, has been shown to suppress the salicylic acid response, PR1, and plant resistance (Van Butselaar and Van Den Ackerveken, 2020). The trade-off between auxin synthesis, related to growth, and salicylic acid accumulation, related to defense, is particularly marked in root tissue (Denancé et al., 2013), which was the focus of this study. The salicylic acid response and the production of PR1 have been previously identified as a key defense indicator differentiating Fusarium wilt resistant and susceptible banana cultivars (Van Den Berg et al., 2007; Wang et al., 2015; Ramu et al., 2016; Li et al., 2017; Zhang et al., 2019, 2020). The exogenous application of salicylic acid has also been demonstrated to induce partial resistance to Fusarium wilt in bananas (Wang et al., 2015; Emilda et al., 2020). Systemic acquired resistance, in parallel with salicylic acid defense is likely controlled by the production of nitric oxide, itself controlled by N nutrition (Sun et al., 2020). Mur et al. (2017) suggest nitric oxide-dependent defense is enhanced by nitrate fertilization and reduced by ammonium. Our findings agree that PR1 expression decreased as more ammonium fertilizer was added to the system, though we did not find a significant increase from nitrate fertilization, nor an N form effect on the “MAPK signaling pathway—plant” pathway. Further examination of the proteins that contribute to PR1 expression would be useful to understand how the full signaling pathways respond to N fertilization.

The expression of PR1 at low fertilization rates is greater with ammonium than with nitrate and the decrease of PR1 in response to ammonium is much greater than the increase in response to nitrate causing the expression rates based on the two N forms to intersect at high rates (Figure 8). This is despite the δ^15^N findings indicating that at low zfertilization rates all plants are likely taking up nitrate (Figures 2, 3) and there is minimal pH difference between the two fertilizer forms (Figure 2). This suggests that a separate mechanism may be responsible and warrants further investigation. There was no statistically significant N form effect on either disease severity or inoculated plant dry weight at low N rates despite the difference in PR1 expression between the two N forms so there may be additional important factors not considered here.

Initiation of defensive signaling in bananas as a response to Foc exposure has been shown to affect the level of banana resistance to Foc (Zhang et al., 2019). The group of proteins categorized under the gene ontology of “Defense response”, which was differentially regulated in inoculated compared with uninoculated samples, is the primary response to the presence of a foreign body or injury (Gene Ontology, 2021). The “Chitin binding” gene ontology also could be a defense-associated response to the fungal pathogen, due to the importance of chitin in fungal cell walls (Zhang et al., 2019). The “Signaling receptor activity” pathway, which includes immune receptor activity, was also affected by Foc inoculation possibly indicating a reduction in the transmission of the defense response (Supplementary Information 3). In agreement with previous studies, inoculation with Foc caused a substantial upregulation of PR1, demonstrating the importance of PR1 to the banana's defense against Foc (Li et al., 2015; Zhang et al., 2019).

The effect of N fertilizer application on disease severity is complicated by a change in soil pH, which affects pathogen abundance and soil bacteria. Soil pH was reduced by the addition of ammonium and slightly increased, though statistically insignificant, by nitrate addition. Nitrate fertilizer has been previously shown to provide protection to plants from Fusarium wilts whereas ammonium typically increases disease severity (Morgan and Timmer, 1984; Wang et al., 2016; Mur et al., 2017; Zhou et al., 2017). However, we found that increased application of both nitrate and ammonium was significantly positively correlated with internal disease severity (Figure 1), suggesting that in our experiment soil pH change may not be the principal determinant of disease severity. Morgan and Timmer (1984) found ammonium and nitrate-treated soil had a final pH of 5.8 and 6.9, respectively. The soils we used had a greater pH buffering capacity, possibly limiting the suppressive potential of pH increase and explaining the disparity between our findings and that of previous researchers.

Our δ^15^N findings provide further insight into the uptake of the two N forms (Figure 3). At low N fertilizer rates, the nitrate-fertilized plants took up less than half of their N as nitrate, directly from the fertilizer (δ^15^N of plants nearer to that of soil than fertilizer) whereas the ammonium-fertilized plants did not take up the equivalent amount of fertilizer-derived ammonium N. Either they took up all their N from the soil N pool or from fertilizer-derived N that had nitrified to nitrate (Figure 2). Therefore, at low N fertilizer rates, plants appeared to take up mostly nitrate, irrespective of the form applied. Conversely, at high fertilizer rates plants drew N from the fertilizer supplied but in a combination of forms despite nitrate concentrations being far higher than ammonium concentrations irrespective of the form applied (Figure 2).

Separating the effect of N rate from the effect of pH decline caused by ammonium on Fusarium wilt disease symptoms and the growth of Foc is difficult (Orr and Nelson, 2018; Segura-Mena et al., 2021). Organisms differ in their capacities to function at low pH. Generally, plants are regarded as having greater tolerance to low pH, fungi moderate tolerance, and bacteria are intolerant to reductions in pH (Rousk et al., 2009, 2010). Soil pH was negatively correlated with soil bacterial alpha diversity (Figure 5) and to a minor extent Foc DNA concentration, indicating that the effect of N on the soil microbiome may be occurring at least partially *via* pH change. However, changes in Foc DNA concentration were unrelated to banana plant disease severity (Figure 4) indicating that Foc abundance was not a driver of disease severity in this case. Our results agree with those of Pittaway et al. (1999) who found pathogen activity was unrelated to disease severity. We inoculated the upper layer of the soil but measured the pathogen population in the rhizosphere where the pathogen and host interact. Growth of the pathogen through the soil exposes the pathogen to the treatments and other members of the disease triangle, resulting in either suppressiveness or conduciveness. The downside of this approach is that the distance between the soil surface and plant root will vary between replicates, adding to variability in the rhizosphere pathogen population. Another limitation of our approach is that we did not measure virulence, which may or may not be linked to abundance. Virulence factors such as Fusaric acid production, or virulence-related gene expression, may have responded to N supply irrespective of abundance (Divon et al., 2006; Bolton and Thomma, 2008; López-Berges et al., 2010; Ding et al., 2018). Li et al. (2019) recently applied simultaneous transcript profiling of Foc and the banana plant during infection. This approach may offer further insight into the effect of N on pathogenicity in the future by directly coupling the response of the plant and pathogen.

Soil pH was not the only environmental factor altered by the nitrogen treatments in this experiment. Most major ions were equalized between treatments, however, the rate of chloride and sodium applied as fertilizer differed between treatments. Despite these differences, it is unlikely that either of these ions was responsible for the treatment effects. There is limited evidence that either chloride or sodium affects Fusarium wilt severity except under high concentrations (Orr and Nelson, 2018). Treatments demonstrating a relationship between Fusarium wilt severity and chloride, sodium or electrical conductivity were orders of magnitude larger than the differences in our fertilizer applications (Woltz et al., 1992; Dominguez et al., 2001; Triky-Dotan et al., 2005).

Inoculation with Foc was significantly associated with changes in the beta diversity of the bacterial (16S) and fungal (ITS) rhizosphere communities and inoculated samples had lower fungal alpha diversity. Contrary to the findings of Effendi et al. (2019), we found that inoculation was not associated with a reduction in the alpha diversity of the bacterial community. Ou et al. (2019) determined that inoculation with Foc increased bacterial alpha diversity in disease suppressive soils and decreased it in conducive soils. The soil we used was relatively disease conducive compared to other soils cultivated for bananas in Australia (Bowen et al., 2019) though there is no way to compare our soils to those of Ou et al. (2019) as conduciveness is a relative measure. It is worth noting that by measuring rhizosphere soil DNA we inherently included residual DNA from organisms that were present prior to inoculation possibly artificially increasing the diversity of the inoculated samples (Carini et al., 2016).

The controlled growing conditions determined in this study differed from commercial situations, and the effects demonstrated the need for field investigation across soil types. However, several previous studies suggest that our findings should be applicable to field situations. For example, a meta-analysis of the effect of N addition on fungal plant pathogens identified no difference between field and pot trials or when the pathogen was naturally vs. artificially introduced (Veresoglou et al., 2013). Additionally, the effect of N on Fusarium wilt severity caused by Race 1 and Tropical Race 4 are similar (Segura-Mena et al., 2021) and infection with these two races initiates a similar defense response (Li et al., 2013). The similarities between Foc Races and agreement between field and pot experiments suggest that the results of our experiment may be applicable to wider conditions though it would be valuable to test this.

## Conclusions

Our results demonstrated that a high rate of N fertilizer led to an increase in the severity of the Fusarium wilt of bananas and suggest this may be partially due to a reduction in the defensive capabilities of the plant. The effect of N form requires further investigation, as PR1, a key defensive compound was not affected by nitrate, despite nitrate fertilizer being positively correlated with disease severity. The increase in disease severity may also have been partially due to a reduction in bacterial diversity caused by a decrease in soil pH following ammonium addition. The abundance of the pathogen in the rhizosphere appeared to have no effect on disease severity but was weakly related to soil pH. It would appear there is a threshold of N application, above which elevated internal disease severity is detrimental to plant dry weight. It may in the future be possible to apply fertilizer to maximize banana plant growth while avoiding the penalty of increased internal Fusarium wilt severity, by enhancing the expression of PR1 or related defense genes, decoupling the plant growth—defense trade-off, and by preventing soil pH decline.

## Data availability statement

The datasets presented in this study can be found in online repositories. The names of the repository/repositories and accession number(s) can be found below: https://massive.ucsd.edu/ProteoSAFe/static/massive.jsp, MSV000089270.

## Author contributions

RO, PD, AP, and PN contributed to the conception and experimental design. RO carried out the trial, collected the samples, and wrote the first draft of the manuscript. PD, YW, DB, HB, and HL-G performed sample analysis and assisted with statistical analysis. MC assisted with statistical analysis. All authors contributed to manuscript revision, read, and approved the submitted version.

## Funding

This project has been funded by Hort Innovation, using the Hort Innovation banana research and development levy, co-investment from Queensland Government and contributions from the Australian Government (Project Code BA14014). Hort Innovation is the grower-owned, not-for-profit research and development corporation for Australian horticulture.

## Conflict of interest

The authors declare that the research was conducted in the absence of any commercial or financial relationships that could be construed as a potential conflict of interest.

## Publisher's note

All claims expressed in this article are solely those of the authors and do not necessarily represent those of their affiliated organizations, or those of the publisher, the editors and the reviewers. Any product that may be evaluated in this article, or claim that may be made by its manufacturer, is not guaranteed or endorsed by the publisher.
