# Supplementary material for: Nitrogen fertilizer rate but not form affects the severity of Fusarium wilt in banana
Source: Front Plant Sci. 2022 Jul 22;13:907819. doi: 10.3389/fpls.2022.907819 (PMC9356348; doi:10.3389/fpls.2022.907819)
Supplement: Supplementary file 1 [file Data_Sheet_1.docx]

Supplementary information 1:

TFA proteomics extraction method for banana roots

1. Weigh approximately 0.016 g of dried sample into 2 mL Eppendorf tube, record exact sample weight.
2. Freeze dry samples at -60℃.
3. Add 300 uL 100% TFA (enough to cover the sample).
4. Agitate at room temperature at ~800 × *g* for 30 minutes.
5. Add 300 uL of saturated Tris base (same volume as TFA).
6. Centrifuge at 13,000 × *g* for 5 minutes.
7. Avoiding the pellet transfer supernatant to new 2 mL centrifuge tube. Repeat steps 6 and 7 twice to remove all particulate matter from the extract.
8. Add 50 uL of stock protein solution into 1.5 mL centrifuge tube.
9. Add 85 uL saturated Tris base, check pH is between 7.5 and 8.5.
10. Make 0.5 M stock solution of TCEP reducing agent for use in samples.
11. Dilute protein solution 1:50 with TCEP for final TCEP concentration of 0.010 M. If using volumes listed above need ~2.7 uL.
12. Agitate at 40℃ at ~800 × *g* for 20 minutes. Add 1:60 ratio of 15% Acrylamide: protein solution for final concentration of 0.025 M Acrylamide.
13. Agitate at 90℃ at 800 × *g* for 3 minutes with weight on the sample caps to prevent popping of caps due to heat.
14. Leave for 20 minutes at room temperature for solutions to cool with weight on lid.
15. Centrifuge for 0.5 minutes at >1000 × *g* to remove precipitation on lid.
16. Test pH of samples is between 7.5 and 8.5.
17. Add 1 ug SOLu-Trypsin (Sigma) to digest proteins. Mix 40 uL Trypsin with 760 uL 50 mM TEAB, then add 20 uL to each sample.
18. Agitate overnight at 39℃. Alternatively can digest for 1 hour with a Barocycler.
19. Add 0.5 ug more SOLu-trypsin (same solution as above).
20. Centrifuge for 0.5 minutes at >1000 × *g* to remove precipitation on lid.
21. Agitate for 120 minutes at 37 ℃.
22. Add TFA to protein solution for 1:5 ratio to give final TFA concentration of 4%.
23. Check pH to make sure it is between 3 and 4.
24. Solid-phase stage tips were prepared by stacking two layers of Empore™ Octadecyl C18 (Supelco/Sigma-Aldrich, USA) disc punches in a 200 uL pipette tip. Stage tips were supported over a 2 mL tube and washed via centrifugation at 1300 × *g* for 2 minutes with 100 uL of methanol followed by 100 uL of 0.2% TFA.
25. Centrifuge samples at 13,000 × *g* for 2 minutes and transfer the supernatant to the stage tip.
26. Centrifuge at 1300 × *g* for 30 minutes until solution has passed through tip into fresh centrifuge tube. Reload the solution back onto the same stage tip and centrifuge at 1300 × *g* for 30 minutes.
27. Wash with 200 uL 0.2% TFA. Centrifuge at 1300 × *g* for 30 minutes 3 times. Take tips to fresh **1.5mL Eppendorf tubes (Pointy ones)**
28. Centrifuge at 1500 × *g* to wash the stage tips into fresh centrifuge tubes with 100 uL 60% Acetonitrile, 0.2% formic acid.
29. Dry on speed vac at room temperature for approximately 1hour, until completely dry.
30. Store the sample at -80℃ until use.

Quantifying protein concentration

1. Re-suspend peptides in 15 uL of 2% Acetonitrile, 0.1% formic acid.
2. Add Biognosys IRT internal standard as per manufacturer specifications and mix the sample.
3. Place 1 uL on Nanodrop (Thermofisher) to quantify protein concentration using method 205
4. Dilute samples with 2% Acetonitrile, 0.1% formic acid and internal standard to the minimum sample peptide concentration or 0.1 ug/uL whichever is higher**.**
5. Transfer samples to 0.5 mL tubes, placed inside a 1.5 mL tube and centrifuge at 13,000 × *g* for 5 minutes.
6. Transfer supernatant into a mass spec vial for analysis.

Supplementary information 2:

The assessment of alpha diversity measures of bacteria (16S) and fungi (ITS) in rhizosphere soil as affected by the three main treatments and interactions.

16S

| Predictor variable | Sobs | | Shannon | | Simpson |  | Chao1 |  | Faith's PD |  |
| --- | --- | --- | --- | --- | --- | --- | --- | --- | --- | --- |
|  | *F (1, 82)* | *P* | *F (1, 82)* | *P* | *F (1, 82)* | *P* | *F (1, 82)* | *P* | *F (1, 82)* | *P* |
| N rate | 27.6 | < 0.001 | 14.1 | < 0.001 | 3.8 | 0.007 | 13.7 | < 0.001 | 32.9 | < 0.001 |
| N form | 0.2 | 0.665 | 0.4 | 0.554 | 0.1 | 0.724 | 0.2 | 0.649 | 0.3 | 0.606 |
| Inoculation | 0.9 | 0.361 | 0.9 | 0.343 | 2.5 | 0.119 | 0.9 | 0.347 | 0.0 | 0.890 |
| N rate * N form | 4.9 | 0.03 | 1.6 | 0.212 | 0.4 | 0.773 | 2.4 | 0.124 | 5.6 | 0.021 |
| N rate * Inoculation | 0.6 | 0.446 | 0.0 | 0.987 | 0.3 | 0.883 | 0.5 | 0.502 | 2.0 | 0.163 |
| N form * Inoculation | 0.2 | 0.677 | 0.0 | 0.900 | 0.2 | 0.625 | 0.5 | 0.476 | 0.0 | 0.969 |
| 3 way interaction | 0.1 | 0.779 | 0.0 | 0.858 | 0.7 | 0.586 | 0.0 | 0.906 | 0.4 | 0.544 |

ITS

| Predictor variable | Sobs | | Shannon | | Simpson |  | Chao1 |  |
| --- | --- | --- | --- | --- | --- | --- | --- | --- |
|  | *F(1, 82)* | *P* | *F(1, 82)* | *P* | *F(1, 82)* | *P* | *F(1, 82)* | *P* |
| N rate | 0.01 | 0.926 | 0 | 0.977 | 2.7 | 0.040 | 4.66 | 0.034 |
| N form | 0 | 0.998 | 0.13 | 0.722 | 1.0 | 0.333 | 2.08 | 0.153 |
| Inoculation | 0 | 0.981 | 4.93 | 0.029 | 32.7 | <0.001 | 3.43 | 0.068 |
| N rate * N form | 0.03 | 0.859 | 0.06 | 0.805 | 1.1 | 0.385 | 0.96 | 0.33 |
| N rate * Inoculation | 0.38 | 0.538 | 0.03 | 0.872 | 0.9 | 0.461 | 4.67 | 0.034 |
| N form * Inoculation | 0.23 | 0.634 | 0 | 0.945 | 1.8 | 0.185 | 2.28 | 0.134 |
| 3 way interaction | 0.01 | 0.94 | 0.29 | 0.589 | 0.3 | 0.875 | 1.31 | 0.256 |

Supplementary information 3:

Differential expression based on inoculation with *Fusarium oxysporum* f.sp. *cubense*.

**Table 1**: Significantly differentially expressed Gene ontologies, based on proteins that are differentially expressed with inoculation with *Fusarium oxysporum* f.sp. *cubense*.

| GO ID | Term | Annotated | DE | *P* | Sub ontology |
| --- | --- | --- | --- | --- | --- |
| GO:0004864 | Protein phosphatase inhibitor activity | 5 | 5 | <0.001 | MF |
| GO:0038023 | Signalling receptor activity | 5 | 5 | <0.001 | MF |
| GO:0010427 | Abscisic acid binding | 5 | 5 | <0.001 | MF |
| GO:0030246 | Carbohydrate binding | 17 | 8 | 0.004 | MF |
| GO:0030145 | Manganese ion binding | 11 | 6 | 0.005 | MF |
| GO:0004553 | Hydrolase activity, hydrolyzing O-glycos... | 40 | 14 | 0.016 | MF |
| GO:0008061 | Chitin binding | 4 | 3 | 0.018 | MF |
| GO:0004448 | Isocitrate dehydrogenase activity | 4 | 3 | 0.018 | MF |
| GO:0004190 | Aspartic-type endopeptidase activity | 11 | 5 | 0.027 | MF |
| GO:0016614 | Oxidoreductase activity, acting on CH-OH... | 29 | 9 | 0.029 | MF |
| GO:0004866 | Endopeptidase inhibitor activity | 7 | 4 | 0.029 | MF |
| GO:0009738 | Abscisic acid-activated signalling pathwa... | 5 | 5 | <0.001 | BP |
| GO:0006952 | Defense response | 10 | 7 | <0.001 | BP |
| GO:0006511 | Ubiquitin-dependent protein catabolic pr... | 19 | 7 | 0.039 | BP |
| GO:0005840 | Ribosome | 112 | 25 | 0.014 | CC |

**Table 2**: Significantly differentially expressed KEGG pathways, based on proteins that are differentially expressed with inoculation with *Fusarium oxysporum* f.sp. *cubense*. Pathways are ordered based on KEGG pathway number, inferring functional similarity.

| K | Function | Measured | DE | *P* |
| --- | --- | --- | --- | --- |
| 03010 | Ribosome | 71 | 22 | 0.014 |
| 00500 | Starch and sucrose metabolism | 14 | 6 | 0.015 |
| 00520 | Amino sugar and nucleotide sugar metabolism | 21 | 8 | 0.019 |
| 00051 | Fructose and mannose metabolism | 9 | 4 | 0.024 |
